# Development of microsatellite loci for two New World vultures (Cathartidae)

**DOI:** 10.1186/s13104-019-4295-z

**Published:** 2019-05-09

**Authors:** Darren J. Wostenberg, Jennifer A. Fike, Sara J. Oyler-McCance, Michael L. Avery, Antoinette J. Piaggio

**Affiliations:** 1 USDA APHIS, National Wildlife Research Center, 4101 Laporte Ave, Fort Collins, CO 80521 USA; 2 U.S. Geological Survey, Fort Collins Science Center, 2150 Centre Ave #C, Fort Collins, CO 80526 USA; 3 USDA APHIS, National Wildlife Research Center, Florida Field Station, 2820 E University Ave, Gainesville, FL 32641 USA

**Keywords:** Black vulture, Turkey vulture, Microsatellites, Next-generation sequencing, Genetic diversity

## Abstract

**Objective:**

Use next-generation sequencing to develop microsatellite loci that will provide the variability necessary for studies of genetic diversity and population connectivity of two New World vulture species.

**Results:**

We characterized 11 microsatellite loci for black vultures (*Coragyps atratus*) and 14 loci for turkey vultures (*Cathartes aura*). These microsatellite loci were grouped into 3 multiplex panels for each species. The number of alleles among black vulture samples ranged from 2 to 11, and 3 to 48 among turkey vulture samples.

**Electronic supplementary material:**

The online version of this article (10.1186/s13104-019-4295-z) contains supplementary material, which is available to authorized users.

## Introduction

New World vultures (Cathartidae) are a monophyletic group that diverged from other bird species 55 million years ago [1]. This group of scavenging-specialized birds provide critical ecosystem services [2], and one genus of this family is critically endangered, California Condor (*Gymnogyps*). Data from the US Breeding Bird Survey indicate that during 2005–2015, turkey vulture (*Cathartes aura*) populations grew by 3.1% annually and black vultures (*Coragyps atratus*) increased 5.9% annually during the same period [3]. These two species are well adapted to human-dominated landscapes [2, 4] and coincident with rising populations have been reports of increased property damage, livestock depredations, and aircraft safety issues associated with vultures [4–6].

Throughout most of their range, black vultures are resident year-round, but they do make irregular short-term movements in response to adverse weather conditions [4]. Turkey vultures are highly migratory with large numbers of wintering birds enlarging local populations in the southern US from late fall to early spring [4]. Both species have expanded their ranges northward over the past 50 years [4]. Using molecular methods to further investigate the ecological characteristics of these species could help inform our understanding of the roles these birds play in their present and expanded range. The high mutation rate and heritability of microsatellites make them ideal markers for studying demographic patterns [7].

Microsatellite loci have been developed for several Old World vulture species [8–11], yet only one New World vulture species, the critically endangered California condor (*Gymnogyps californianus*) [12]. Therefore, our goal was to use next-generation sequencing to develop microsatellite panels for two broad-ranging, genetically-diverse species of New World vultures, turkey vultures and black vultures. Development of species-specific markers reduces the risk of ascertainment bias that can occur when applying markers developed in other species [13]. These markers could be used to reliably assess genetic diversity, population connectivity, relatedness among individuals, demographic parameters, and population boundaries for black and turkey vultures. In concert with ecological field studies such as satellite telemetry studies, this information can be used to better understand the ecology of black and turkey vultures across their ranges.

## Main text

### Methods

Tissue samples (muscle) for microsatellite primer development were collected from black vultures and turkey vultures from control efforts in Alachua County, Florida in April 2004 and February 2011, respectively. Control efforts were performed on military bases and conducted by the USDA, Wildlife Services, National Wildlife Research Center personnel to reduce collisions between vultures and military aircraft. DNA for shotgun genome sequencing and primer development was isolated from one tissue sample from each species using a modified ammonium acetate protocol (Gentra Puregene Kit QIAGEN, Hilden, Germany). Shotgun sequencing libraries were developed using a Nextera DNA Sample Preparation Kit (Illumina, San Diego, California, USA) following the manufacturer’s instructions. Libraries were pooled and sequenced in one 300 bp PE MiSeq v.3 (Illumina) run. The Perl script PAL_FINDER_v0.02.03 [14], which uses Primer3 [15] for primer design, was used to identify and design primers for potential microsatellite loci. The MiSeq run FASTA files were submitted to GenBank (SRA accession: PRJNA498072).

Samples used to evaluate amplification and heterozygosity of selected primer pairs were obtained from single populations of black vultures (*n *= 30) and turkey vultures (*n *= 30). Black vulture blood samples were collected at Everglades National Park, Florida in November and December 2012. Turkey vulture blood samples were collected at Key West Naval Air Station, Florida in January and February 2013. DNA was isolated from the blood samples using the DNeasy Blood & Tissue Kit and QIAcube robotic workstations (QIAGEN). M13 primer sequences were added to the 5′ end of the forward primer in each primer pair. PCRs were performed in a 9-µL reaction using 1.0 µL of template DNA, 0.2 µL GoTaq^®^ Flexi DNA polymerase (Promega, Madison, Wisconsin, USA), 2.0 µL 5× buffer (Promega), 1.0 µL dNTP (Promega), 1.0 µL MgCl_2_ 25 mM (Promega), 1.0 µL of each 10 µM primer, and 1.0 µL of 1 µM M13 primer labeled with 6-FAM. All reactions were amplified on a Mastercycler ep Gradient thermal cycler (Eppendorf, Hamburg, Germany) using the thermal profile initial denaturation at 94 °C for 5 min followed by 35 cycles of 94 °C for 30 s, 58 °C for 45 s, and 72 °C for 45 s. Cycling was followed with a final extension for 5 min at 60 °C. PCR products were added to a mixture of HiDi Formamide (Life Technologies, Carlsbad, California, USA) and GeneScan 500 LIZ Size Standard (Life Technologies). Samples were run on an ABI 3500 genetic analyzer (Life Technologies) and loci performance was manually evaluated using GeneMapper v.5.0 (Life Technologies).

Loci were organized into three panels based on PCR product size range and primer complementarity using Multiplex Manager v.1.2 [16] for each species (Tables 1, 2, 3). Forward primers were ordered from Applied Biosystems (Life Technologies) with the 5′ fluorescent dyes 6-FAM, VIC, NED, and PET and PIG-tails [17] added to the 5′ end of the reverse primer to reduce stutter (Tables 1, 2). PCR chemistry and primer volumes were optimized for each panel using QIAGEN 2× Multiplex PCR Master Mix and 1.0 µL of template DNA. All reactions were amplified on an Eppendorf Mastercycler thermal cycler using the thermal profile: initial denaturation at 94 °C for 5 min followed by panel-specific number of cycles (Table 3) of 94 °C for 30 s, 58 °C for 45 s, and 72 °C for 45 s. Cycling was followed with a final extension for 5 min at 60 °C. Samples were genotyped as described above.

**Table 1:** Primer sequences, motifs, and characteristics of the 11 microsatellite loci developed and optimized from the black vulture (*Coragyps atratus*)

Locus	Panel	Dye label	Primer sequence (5′–3′); F, forward; R, reverse	Repeat motif	*n*	Size range (bp)	*N*_A_	*H*_O_	*H*_E_
BLVU-36	A	6-FAM	F: CTGAACGGAAACAGAGCTGC	AAAG_(7)_ AACG_(6)_	30	223–242	4	0.68	0.66
			R: CACTATGACCCCTTATGACTCTGG						
BLVU-11^a,b^	A	VIC	F: CTTGAAGAGCAAAGTCGGGG	AGT_(13)_ TTC_(11)_	30	225–237	3	0.10	0.42
			R: AGGACAAATGTGCCTTTCGG						
BLVU-37	A	PET	F: CTAATGGCTCCAGACCCAGG	TATC_(12)_	30	258–266	3	0.52	0.46
			R: TTTTGTCCACCTCCTGTCCC						
BLVU-05^a,b^	B	6-FAM	F: GACCTATCCACATGAATGCC	GA_(37)_ AG_(17)_	30	295–317	9	0.32	0.64
			R: GCCTCTGTTAGTATTCCATCCCC						
BLVU-38^a,b^	B	6-FAM	F: TGTCACCTGGAGCTCTGTCC	ATCT_(13)_	30	168–188	5	0.29	0.62
			R: TCATTAGCATGAAATGAAGGTGC						
BLVU-09	B	VIC	F: CCTCCATAGATGTGCCCTAACC	GAAA_(12)_ AAGG_(18)_	30	272–320	5	0.39	0.39
			R: ACAGCTTCTCCCTGTGTCCC						
BLVU-18	B	PET	F: CTCTCTCTAACCGGCTCTACGC	GTT_(10)_	30	117–123	2	0.06	0.06
			R: GAAGAAGAGAGAGGCGGCG						
BLVU-33	C	6-FAM	F: GGGTAGCAAGAGAAAGAGGGG	AGAC_(6)_ GGAA_(7)_	30	350–406	11	0.71	0.84
			R: ATTGTGCATTTCCTCCTGGC						
BLVU-39	C	VIC	F: CTTCCTTCCTCTGCCTGC	TGCC_(14)_	30	109–129	5	0.74	0.70
			R: TGAACAGGACTTGATTGTCTCC						
BLVU-40	C	NED	F: CCTCTATTGGCTTCAGCAGG	TTCC_(8)_	30	274–282	3	0.45	0.50
			R: GCAAGAGGAAGAGTGGAAGG						
BLVU-27	C	PET	F: CCAAAACCTGCCACTGTCC	AAAT_(12)_	30	214–226	3	0.65	0.59
			R: GGTGACATTTTAATGCTGGGC						

*n* is the sample size, *N*_A_ is the number of alleles, *H*_O_ is the observed heterozygosity, and *H*_E_ is the expected heterozygosity

^a^Showed significant deviation from Hardy–Weinberg equilibrium after Bonferroni corrections [20]

^b^Showed evidence of null alleles

**Table 2:** Primer sequences, motifs, and characteristics of the 14 microsatellite loci developed and optimized from the turkey vulture (*Cathartes aura*)

Locus	Panel	Dye label	Primer sequence (5′–3′); F, forward; R, reverse	Repeat motif	*n*	Allele size range (bp)	*N*_A_	*H*_O_	*H*_E_
TUVU-21^b^	A	6-FAM	F: TTGTTTGGCTCCATGTTTGG	CT_(10)_ AC_(8)_ AC_(10)_	30	202–210	3	0.20	0.39
			R: ACACCCATTCAAATGCAAGC						
TUVU-06	A	6-FAM	F: GAGTCAGCAATGGTGGTTGC	GAA_(25)_ GAA_(8)_	30	326–533	17	0.77	0.83
			R: ACTGTAGCAGTGACGGCAGC						
TUVU-31	A	VIC	F: AAGTAAATAGCTGTCTAACTGTTCATCC	TG_(15)_ AT_(8)_	30	122–152	8	0.73	0.81
			R: CTTTCATGCCTTGATTTCCC						
TUVU-23	A	NED	F: GAAACGGTATTTGCCTTGCC	TTCC_(12)_	30	155–161	6	0.60	0.60
			R: AAAACTCCAAGGGGAGGAGG						
TUVU-39^b^	A	PET	F: GGTTCAGGTGAGAGAAACCCC	AAAG_(23)_	30	188–311	20	0.77	0.90
			R: GAAAACCCCTCTGGGAAACC						
TUVU-14^a,b^	B	6-FAM	F: CCTAGTCCGGAAACACAGGG	ATT_(14)_ ATT_(6)_	30	347–371	7	0.50	0.75
			R: TTAAACTGAAATGTGTGAAGAAGCG						
TUVU-07	B	6-FAM	F: TGGGATGTGAAGGAGAACAGC	GGAA_(28)_	30	160–226	15	0.87	0.92
			R: TGACTCCTGTACAAAATTAGATCCTTCC						
TUVU-45	B	VIC	F: AATAATCCATGAGCACCAGGC	GTTT_(14)_	30	131–158	7	0.50	0.45
			R: CCCATAAACTCAAGCATTGGC						
TUVU-03	B	PET	F: AGGTTCATTAGCAGAGGCGG	AAAG_(9)_ AAAG_(18)_	30	261–375	30	1.00	0.96
			R: GTGGCAGAAAGAAGCTGAAGG						
TUVU-01	C	6-FAM	F: TCATACACTGGTCGTTCGCC	TC_(6)_ CTTT_(24)_ CTTT_(12)_	30	290–640	47	1.00	0.99
			R: ATTGAAATGCCCTACAGACGG						
TUVU-18	C	VIC	F: GGTTCTGCTGATTTCAACTTTGC	TAA_(11)_ TGA_(9)_	30	240–249	4	0.50	0.47
			R: TTCACCACAGGAAACCAAAGC						
TUVU-37	C	NED	F: GCTGGTTTTGAACAGTGAGGG	AAAG_(27)_ AAAG_(8)_	30	263–415	34	0.93	0.97
			R: TTACAGGTGGGGAATCTCAGG						
TUVU-33	C	PET	F: GCAAATCAGCCTCTGGTGG	TA_(13)_ TA_(6)_	30	330–339	9	0.77	0.83
			R: TTAACTTGGAGGCCAGGAGG						
TUVU-36^a,b^	C	PET	F: CACACGCACACAATGCACC	GC_(8)_	30	169–173	3	0.07	0.45
			R: CACTGCGCGAGTGTGAGG						

*n* is the sample size, *N*_A_ is the number of alleles, *H*_O_ is the observed heterozygosity, and *H*_E_ is the expected heterozygosity

^a^Showed significant deviation from Hardy–Weinberg equilibrium after Bonferroni corrections [20]

^b^Showed evidence of null alleles

**Table 3:** Multiplex PCR characteristics for the three optimized black vulture (*Coragyps atratus*) microsatellite panels (BLVU) and the three optimized from the turkey vulture (*Cathartes aura*) microsatellite panels (TUVU)

Panel	Reaction volume (µL)	*T*_a_ (°C)	No. of cycles	Panel	Reaction volume (µL)	*T*_a_ (°C)	No. of cycles
BLVU-A	10.0	58	32	TUVU-A	13.0	58	34
BLVU-B	9.0	58	32	TUVU-B	16.0	58	37
BLVU-C	9.0	58	32	TUVU-C	15.0	58	44

*T*_a_ is the annealing temperature and no. of cycles is the number of amplification cycles in the thermal cycler protocol

Loci were tested for Hardy–Weinberg equilibrium (HWE) and linkage disequilibrium with observed and expected heterozygosity calculated using Arlequin v.3.5 [18]. Microsatellite loci were checked for the presence of null alleles using Micro-Checker v.2.2.3 [19].

Microsatellite loci designed for each species were tested for PCR amplification in the opposite vulture species (BLVU markers with turkey vulture DNA samples and TUVU markers with black vulture DNA samples). Ten samples were used to test for amplification in each species. Cross-species PCR amplification was performed at the optimized PCR conditions for the target species.

### Results

The MiSeq run generated 9,772,498 (2 × 4,886,249) reads for black vultures and PAL_FINDER identified 59,440 reads with microsatellites. Primer pairs were selected randomly and targeted di-nucleotide, tri-nucleotide, and tetra-nucleotide repeats with minimal complexity in the repeat regions. Based on cost limitations, 58 black vulture microsatellite loci were tested for amplification and polymorphism. Of these 11 loci were identified as polymorphic and amplified consistently. For these 11 loci, the number of alleles ranged from 2 to 11, observed heterozygosity ranged from 0.06 to 0.74, and the expected heterozygosity ranged from 0.06 to 0.84 (Table 1). After Bonferroni corrections [20], three loci showed significant deviation from HWE (BLVU-05, BLVU-11, and BLVU-38) and no loci showed significant evidence of linkage. Estimates of heterozygote deficiency used to detect the presence of null alleles in microsatellite data [19, 21, 22] indicated the presence of null alleles in the black vulture loci BLVU-05, BLVU-11, and BLVU-38.

The MiSeq run generated 9,248,148 (2 × 4,624,074) reads for turkey vultures and PAL_FINDER identified 60,353 reads with microsatellites. Primer pairs for 59 turkey vulture microsatellite loci were tested for amplification and polymorphism. Of these 14 loci were identified as polymorphic and amplified consistently. For these 14 loci, the number of alleles ranged from 3 to 47, observed heterozygosity ranged from 0.07 to 1.00, and the expected heterozygosity ranged from 0.39 to 0.99 (Table 2). After Bonferroni corrections [20], 2 loci showed significant deviation from HWE (TUVU-14 and TUVU-36) and no loci showed significant evidence of linkage. Null alleles were detected in the turkey vulture loci TUVU-14, TUVU-21, TUVU-36, and TUVU-39.

The majority of loci for each species amplified when tested using template DNA from the opposite species under optimized PCR conditions. Eight of 11 (72.7%) BLVU loci amplified using turkey vulture DNA samples, and 10 of 14 (71.4%) TUVU loci amplified using black vulture DNA samples (Additional file 1: Tables S1, S2). Five loci reliably amplified in both species (*n* = 10 of each species) and produced unique allelic distributions for each species and could thus be used to separate the species and identify each uniquely in a single multiplexed PCR panel (Additional file 1: Table S3).

### Discussion

The development of these microsatellite loci provides a new tool for studying New World vulture populations. Data generated from these markers could help better understand vulture demography, population structure, and relationships among individuals at a roost site. While both sets of microsatellite loci were variable for the samples tested, the TUVU loci included multiple hypervariable loci (loci with ≥ 20 alleles: TUVU-39, 20 alleles; TUVU-03, 30 alleles; TUVU-37, 34 alleles; and TUVU-01, 47 alleles; Table 2). Not considering these four markers, the remaining 10 TUVU loci averaged 7.9 alleles per marker. The BLVU loci averaged 4.8 alleles across 11 markers.

The evaluation of amplification of markers developed in one species in the other species (e.g. black vulture markers tested in turkey vultures) revealed that at least half of each set of loci generate PCR products in the other species at the optimized PCR conditions, however only a few loci from each group performed particularly well. Among the BLVU loci, BLVU-09, BLVU-18, BLVU-27, BLVU-33, BLVU-36, and BLVU-39 generated clear polymorphic chromatograms with distinct alleles and could be used in addition to the turkey vulture microsatellite panels. Among the TUVU loci, TUVU-18, TUVU-21, TUVU-31, TUVU-33, and TUVU-45 generated clear polymorphic chromatograms with distinct alleles in black vultures.

These microsatellite markers represent a robust subset of possible markers identified by our genome sequencing results. Financial limitations and planned studies for these markers resulted in the identified markers being sufficient for our purposes. However, for other researchers with time and resources to pursue other markers, we have provided a summary of our PAL_FINDER results for each species (Additional files 2, 3). We have identified the primer combinations we tested and made comments regarding their performance. In the meantime, we have provided a set of markers that are polymorphic, perform reliably, and thus can be used for population genetics studies of these two New World vulture species.

## Limitations

- Many of the black vulture loci have only a few alleles so more loci might be needed for some research questions.
- Microsatellites are neutral markers, so they are not useful when investigating adaptation/selection.
- We did not spend much time optimizing loci; we only used ones that worked in initial screenings. Therefore, we may have excluded loci that would work and be polymorphic with more effort.

## Additional files

**Additional file 1: Table S1.** Summary of cross-species PCR amplification of black vulture (*Coragyps atratus*) microsatellite primers tested on turkey vulture (*Cathartes aura*) samples under optimized PCR conditions. Table heading abbreviations are *n* is the sample size and *N*_A_ is the number of alleles. **Table S2.** Summary of cross-species PCR amplification of turkey vulture (*Cathartes aura*) microsatellite primers tested on black vulture (*Coragyps atratus*) samples under optimized PCR conditions. Table heading abbreviations are *n* is the sample size and *N*_A_ is the number of alleles. **Table S3.** A potential panel of markers that can provide species identification between BLVU and TUVU. *N*_A_ is the number of alleles and *T*_A_ is the annealing temperature for the primer pair. Dye is the fluorophore for the marker pair to be visualized on an ABI3500 genetic analyzer.

**Additional file 2.** PAL_FINDER results for black vulture (*Coragyps atratus*) next-generation sequencing run on Illumina MiSeq. MiSeq run files were submitted to the National Center for Biotechnology Information Sequence Read Archive (accession number PRJNA498072).

**Additional file 3.** PAL_FINDER results for turkey vulture (*Cathartes aura*) next-generation sequencing run on Illumina MiSeq. MiSeq run files were submitted to the National Center for Biotechnology Information Sequence Read Archive (accession number PRJNA498072).

## Data Availability

The MiSeq run FASTA files were submitted to GenBank (SRA accession: PRJNA498072). A summary of our PAL_FINDER results for each species can be found in Additional files 2, 3.

## Abbreviations

PCR: polymerase chain reaction
bp: base pair
PE: paired end
BLVU: black vulture
TUVU: turkey vulture
HWE: Hardy–Weinberg equilibrium
N_A_: number of alleles
H_O_: observed heterozygosity
H_E_: expected heterozygosity
T_a_: annealing temperature
