# Supplementary material for: Development of microsatellite loci for two New World vultures (Cathartidae)
Source: BMC Res Notes. 2019 May 9;12:257. doi: 10.1186/s13104-019-4295-z (PMC6506951; doi:10.1186/s13104-019-4295-z)
Supplement: Supplementary file 1 — Additional file 1: Table S1. Summary of cross-species PCR amplification of black vulture (Coragyps atratus) microsatellite primers tested on turkey vulture (Cathartes aura) samples under optimized PCR conditions. Table heading abbreviations are n is the sample size and NA is the number of alleles. Table S2. Summary of cross-species PCR amplification of turkey vulture (Cathartes aura) microsatellite primers tested on black vulture (Coragyps atratus) samples under optimized PCR conditions. Table heading abbreviations are n is the sample size and NA is the number of alleles. Table S3. A potential panel of markers that can provide species identification between BLVU and TUVU. NA is the number of alleles and TA is the annealing temperature for the primer pair. Dye is the fluorophore for the marker pair to be visualized on an ABI3500 genetic analyzer. [file 13104_2019_4295_MOESM1_ESM.docx]

Table S1. Summary of cross-species PCR amplification of black vulture (*Coragyps atratus*) microsatellite primers tested on turkey vulture (*Cathartes aura*) samples under optimized PCR conditions. Table heading abbreviations are *n* is the sample size and *N*_A_ is the number of alleles.

| Locus | *n* | Size range (bp) | *N*_A_ |
| --- | --- | --- | --- |
| BLVU-36 | 10 | 234-260 | 7 |
| BLVU-11 | 10 | - | - |
| BLVU-37 | 10 | 254-260 | 4 |
| BLVU-05 | 10 | - | - |
| BLVU-38 | 10 | 189 | 1 |
| BLVU-09 | 10 | 160-206 | 9 |
| BLVU-18 | 10 | 110-126 | 4 |
| BLVU-33 | 10 | 328-336 | 3 |
| BLVU-39 | 10 | 105-117 | 4 |
| BLVU-40 | 10 | - | - |
| BLVU-27 | 10 | 206 | 1 |

Table S2. Summary of cross-species PCR amplification of turkey vulture (*Cathartes aura*) microsatellite primers tested on black vulture (*Coragyps atratus*) samples under optimized PCR conditions. Table heading abbreviations are *n* is the sample size and *N*_A_ is the number of alleles.

| Locus | *n* | Size range (bp) | *N*_A_ |
| --- | --- | --- | --- |
| TUVU-21 | 10 | 179-180 | 2 |
| TUVU-06 | 10 | - | - |
| TUVU-31 | 10 | 93 | 1 |
| TUVU-23 | 10 | - | - |
| TUVU-39 | 10 | 153 | 1 |
| TUVU-14 | 10 | - | - |
| TUVU-07 | 10 | - | - |
| TUVU-45 | 10 | 131-135 | 2 |
| TUVU-03 | 10 | 79-83 | 2 |
| TUVU-01 | 10 | 487-596 | 11 |
| TUVU-18 | 10 | 210-216 | 3 |
| TUVU-37 | 10 | 181-188 | 3 |
| TUVU-33 | 10 | 331-332 | 2 |
| TUVU-36 | 10 | 175-179 | 2 |

Table S3. A potential panel of markers that can provide species identification between BLVU and TUVU. *N*_A_ is the number of alleles and *T*_A_ is the annealing temperature for the primer pair. Dye is the fluorophore for the marker pair to be visualized on an ABI3500 genetic analyzer.

| Locus | *N*_A_ BLVU | range | *N*_A_ TUVU | range | *T*_A_ | Dye | Cycles | Motif |
| --- | --- | --- | --- | --- | --- | --- | --- | --- |
| BLVU-09 | 6 | 272-315 | 9 | 160-206 | 58 | NED | 32 | tetra |
| BLVU-27 | 4 | 214-230 | 1 | 206 | 58 | PET | 32 | tetra |
| TUVU-21 | 2 | 179-180 | 5 | 202-251 | 58 | FAM | 34 | di |
| TUVU-31 | 1 | 93 | 14 | 122-150 | 58 | VIC | 34 | di |
| TUVU-18 | 3 | 210-216 | 6 | 231-249 | 58 | VIC | 44 | tri |
